# Baylisascariasis (Raccoon Roundworm Infection) in Two Unrelated Children — Los Angeles County, California, 2024

**DOI:** 10.15585/mmwr.mm7428a1

**Published:** 2025-07-31

**Authors:** Aisling M. Vaughan, Dina Kamel, Michelle Chang, Laura Saucier, Susan P. Montgomery, Elizabeth Wendt, Alicia H. Chang, Shamim Islam, Aaron Nagiel, Betty Situ, Jamie Middleton, Dawn Terashita, Sharon Balter, Joy E. Gibson, Jemma Alarcón

**Affiliations:** ^1^Epidemic Intelligence Service, CDC; ^2^Acute Communicable Disease Control Program, Los Angeles County Department of Public Health, Los Angeles, California; ^3^Children’s Hospital Los Angeles, Los Angeles, California; ^4^Veterinary Public Health Program, Los Angeles County Department of Public Health, Los Angeles, California; ^5^Division of Parasitic Diseases and Malaria, National Center for Emerging and Zoonotic Infectious Diseases, CDC; ^6^Community and Field Services Division, Los Angeles County Department of Public Health, Los Angeles, California; ^7^Roski Eye Institute, Department of Ophthalmology, Keck School of Medicine, University of Southern California, Los Angeles, California; ^8^Vision Center, Department of Surgery, Children’s Hospital Los Angeles, Los Angeles, California.

**Summary**

**What is already known about this topic?**

*Baylisascaris procyonis*, a roundworm parasite commonly found in raccoons, can cause baylisascariasis, a potentially severe disease in humans.

**What is added by this report?**

In 2024, two baylisascariasis cases characterized by encephalopathy, ocular larva migrans, peripheral and cerebrospinal fluid eosinophilia, and brain imaging abnormalities were diagnosed in unrelated children in Los Angeles County, California, associated with ingestion of raccoon feces and soil potentially contaminated with *B*. *procyonis.* A substantial delay in diagnosis for one patient led to severe neurologic sequelae.

**What are the implications for public health practice?**

Health care providers should suspect *B. procyonis* infection in patients with eosinophilic meningoencephalitis, especially young children or persons with developmental disabilities or pica and consider empiric treatment with albendazole. The public should avoid contact with raccoons and their feces.

## Abstract

*Baylisascaris procyonis* (raccoon roundworm), a parasite commonly found in raccoons (*Procyon lotor*), can cause severe disease in humans when it invades visceral organs or the ocular and central nervous systems. Without prompt treatment, *B. procyonis* infection can lead to serious complications and death. During September 2024, the Los Angeles County Department of Public Health was notified of two unrelated pediatric patients with neurologic signs and symptoms consistent with baylisascariasis, including behavioral change, lethargy, and gait instability. The first case occurred in an adolescent aged 14 years who had received a previous diagnosis of autism spectrum disorder and had a history of pica (i.e., ingestion of nonfood items); the second case occurred in a previously healthy child aged 15 months. Both were treated with albendazole and corticosteroids. The first patient returned to baseline neurologic status, but delays in diagnosis and treatment of the second patient resulted in severe neurologic sequelae. Epidemiologic investigations identified raccoon feces that had fallen from a rooftop latrine (i.e., a communal raccoon defecation site) as the possible source of exposure for the adolescent. No source of exposure was identified for the younger child. *B. procyonis* infection should be suspected and prompt treatment considered in patients with neurologic symptoms and cerebrospinal fluid or peripheral blood eosinophilia (>1,000 eosinophils/mL of blood), especially young children or persons with developmental disabilities or pica. In addition, the public should be aware of exposure prevention strategies, including preventing raccoon activity around properties, avoiding exposure to raccoon feces, and safely removing raccoon latrines.

## Introduction

*Baylisascaris procyonis* (raccoon roundworm) is an intestinal parasite that causes widespread, typically asymptomatic infection in raccoons (*Procyon lotor*) in the United States (*1*,*2*), where up to 80% of raccoons in the Northeast, Midwest, and West Coast regions are affected (*3*). Raccoons, the primary definitive host of *B. procyonis*, can shed millions of roundworm eggs in their feces every day. Eggs become infective after 2–4 weeks in the environment and can survive for years. When consumed by nondefinitive hosts, the eggs develop into larvae and migrate through body tissues (*1*). Human cases are rare: only 35 cases have been reported in the United States (*1*,*2**,**4*). Most patients have been young and male with developmental disabilities and pica, conditions associated with compulsive consumption of nonfood items such as soil (*5*). Serious complications include prolonged migration and persistence of helminth larvae in the viscera (visceral larva migrans), the brain (neural larva migrans), and the eye (ocular larva migrans); some cases have been fatal. No vaccine to prevent baylisascariasis exists. In cases in which suspicion of exposure is high (e.g., known oral exposure to raccoon feces), treatment with oral albendazole (25–50 mg/kg per day for 10–20 days) might be appropriate, and should be initiated as soon as possible after ingestion of infectious material, ideally within three days (*6*).

Two cases of *B. procyonis* infection in children were reported to the Los Angeles County Department of Public Health (LACDPH) in 2024. One case occurred in an adolescent aged 14 years who had received a previous diagnosis of autism spectrum disorder and had a history of pica; the second case occurred in a previously healthy child aged 15 months. Both patients experienced neurologic changes and were found to have eosinophilic meningitis. This report describes these cases and the public health response efforts to reduce the risk to the population and improve awareness of baylisascariasis among health care providers and the public.

## Investigation and Results

On September 4, 2024, LACDPH was notified by a physician at Children’s Hospital Los Angeles of two possible cases of *B. procyonis* infection in an adolescent (patient A) and a young child (patient B). The families of both patients consented to these cases being described. This activity was reviewed by CDC, deemed not research, and was conducted consistent with applicable federal law and CDC policy.^†^

### Patient A Characteristics, Treatment, and Outcome

**FIGURE 1:**
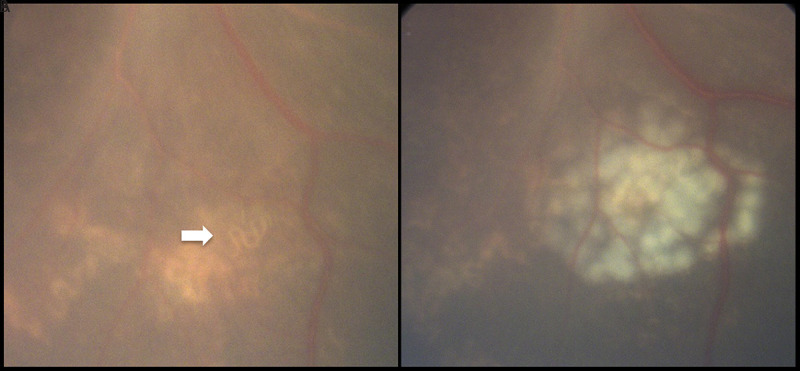
Fundus of patient A’s eye, illustrating a live subretinal nematode (A), and immediately after laser ablation treatment (B) — Los Angeles County, California, 2024 Photos/Vision Center, Department of Surgery, Children’s Hospital Los Angeles

Patient A was an adolescent boy aged 14 years who had received a previous diagnosis of autism spectrum disorder and had a history of pica. In May 2024, he was hospitalized after a week of behavioral changes, including sleepiness, decreased activity, confusion, and unsteady gait (*3*). The results of laboratory analyses of specimens collected during hospital admission indicated mild peripheral eosinophilia (14% eosinophils; >1,000 eosinophils/mL of blood). His symptoms progressively worsened, and brain magnetic resonance imaging (MRI) showed numerous enhancing lesions. Analysis of a lumbar puncture specimen revealed the presence of 15% eosinophils in the cerebrospinal fluid (CSF). The combination of the patient’s symptoms, MRI findings, and especially, peripheral eosinophilia and eosinophilic meningitis prompted concern for *B. procyonis* infection by a clinician who had previously encountered a case. Ophthalmologic examination revealed a live parasitic nematode in the eye, which was treated using laser ablation (Figure 1), leading to a presumptive diagnosis of baylisascariasis.

CDC was consulted for guidance regarding testing and treatment for suspected *B. procyonis* infection. Treatment with 6 weeks of albendazole and corticosteroids was initiated immediately (*6*). CSF and serum specimens were sent to CDC for confirmatory testing by immunoblot assay using the recombinant *B. procynonis* antigen BpRAG1. Serum was weakly positive for *B. procyonis* antibodies, whereas CSF test results were negative; this pattern has been reported previously (*4*). Cross-reactivity with sera from patients with toxocariasis was not observed. The patient’s signs and symptoms subsequently resolved, and he returned to his baseline neurologic status.

### Patient B Characteristics, Treatment, and Outcome

Patient B was a child aged 15 months who had been meeting all his developmental milestones. He was hospitalized in June 2024 for evaluation of encephalopathy, lethargy, generalized weakness, gait instability, and progressively increasing muscle tone. Initial laboratory testing demonstrated peripheral blood eosinophilia (53% eosinophils) and eosinophilic CSF pleocytosis (16%). Brain MRI demonstrated diffuse white matter abnormalities. The child’s initial treatment occurred at two children’s hospitals and included empiric treatment of suspected acute disseminated encephalomyelitis, comprising intravenous immunoglobulin, corticosteroids, the monoclonal antibody rituximab, and plasmapheresis. After 6 weeks, the patient was discharged from the hospital with a gastrostomy tube, a plan to taper the corticosteroids dosage, and scheduled outpatient follow-up appointments. Three months after symptom onset, the patient was taken to an ambulatory neuroimmunology clinic at a third hospital where he was examined by the same clinician who had treated patient A. Similar to patient A, the records indicated marked peripheral eosinophilia and eosinophilic meningitis, raising concern for baylisascariasis. Ophthalmologic evaluation revealed a live parasitic nematode, which was treated by laser ablation. The patient also was treated with 6 weeks of albendazole and corticosteroids. Testing was performed at CDC and as was observed with the first patient, serum was weakly positive for *B. procyonis* antibodies, whereas CSF test results were negative. The patient has persistent, severe neurologic sequelae, including cognitive, motor, and visual impairments.

## Public Health Response

### Site Visits to Residences of Patients A and B

**FIGURE 2:**
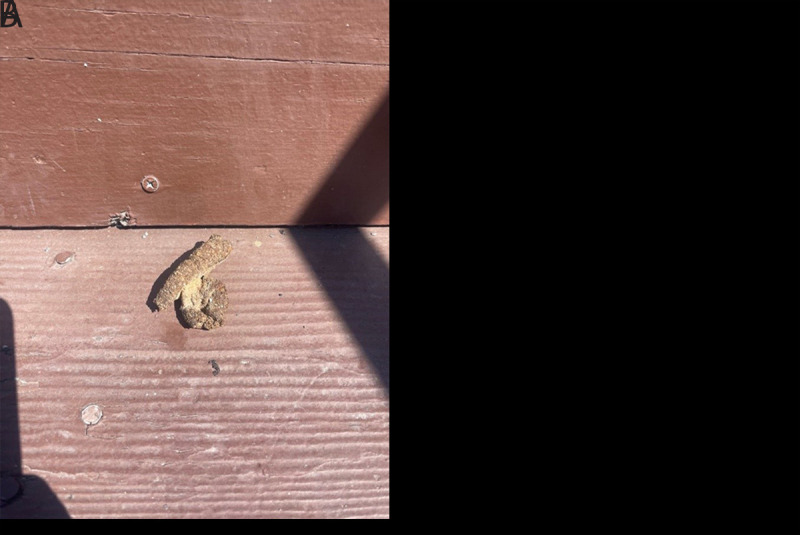
Raccoon latrine on the sloping rooftop of patient A’s home (A) and raccoon feces that fell from the rooftop onto the stairwell leading from the back door (B) — Los Angeles County, California, 2024 Photos/Los Angeles County Department of Public Health

After being notified about these cases, LACDPH immediately initiated public and veterinary health investigations at the patients’ households to identify potential sources of exposure to *B. procyonis*. The family of patient A reported frequent sightings of numerous raccoons near the property and noted that neighbors regularly left food out for community cats, which can attract raccoons. A pet dog was also present at the property.^§^ A raccoon latrine was identified on the sloping rooftop of the property (Figure 2) directly above the entrance to the premises, allowing feces to roll onto the landing below. Dried feces were found below the latrine on the staircase leading from the back door (Figure 2); patient A was known to have pica, and was suspected to have picked up and consumed the feces or feces-contaminated soil at this location. LACDPH recommended immediate removal of the latrine, decontamination of the area, and implementation of raccoon exclusion measures to prevent additional exposure (*7*). However, efforts to mitigate raccoon latrines have been unsuccessful, and the raccoons continue to be observed around the property. Because of the ongoing risk for infection, the treating physician, in consultation with the family, elected to administer a 10-day course of albendazole prophylaxis to two of the patient’s siblings (*6*), one of whom also had pica. In addition, because of the ongoing raccoon infestation despite efforts to deter the raccoons from the property, the family was making plans to move to a new residence.

A site visit to the residence of patient B revealed no evidence of raccoon activity or raccoon latrines, although raccoons were known to be present in the neighborhood. The family mentioned that the child had placed soil and bark mulch into his mouth on multiple occasions at their residence, local parks, and the beach. The family had no pets. A site visit to the child care center attended by patient B also found no evidence of raccoon activity, raccoon latrines, or sandboxes where raccoons might have defecated, and no other children were reported with similar symptoms at the facility.

### Implementation of Public Awareness Campaign

In response to these two cases of baylisascariasis, LACDPH took immediate action to raise awareness regarding preventive strategies to reduce the risk for exposure. A public awareness campaign was conducted at households within an eight-block radius of patient A’s residence, where raccoon feces had been found, as well as at child care centers in the wider area. A total of 476 households and 35 child care centers were contacted. Educational materials were distributed that described the disease and outlined preventive measures, including avoiding feeding wildlife and keeping open food sources inaccessible to community cats and raccoons^¶^ (*7*).

LACDPH also published a press release and webpage to increase public awareness about the risks associated with *B. procyonis* infection in Los Angeles County and how to prevent exposure (*7*). A Los Angeles County Health Alert was published to help guide health care providers through the clinical presentation, the importance of early identification, diagnosis, timely treatment, and reporting of *B. procyonis* infections (*4*). Finally, an Animal Health Advisory was issued to veterinarians regarding *B. procyonis* transmission to domestic pets, particularly dogs, which can serve as definitive hosts, that also provided guidance about prevention strategies and safe removal of raccoon latrines (*8*).

## Discussion

This report describes the public health response to two cases of *B. procyonis* eosinophilic meningoencephalitis in unrelated pediatric patients reported during a short period (September 2024) in Los Angeles County, California. Few cases of *B. procyonis* eosinophilic meningoencephalitis have been documented in the United States (*5*,*9*), and most of these patients have been young, male children with developmental disabilities, often with pica; however, infections have occurred in older age groups and in persons with occupational exposure (*5*). Despite its severe adverse health effects, the true incidence of *B. procyonis* infection in humans is not well understood. A recent serologic study in Santa Barbara County, California found a 7% seroprevalence in the general population (*10*), suggesting that asymptomatic or subclinical human infections are more common than previously thought; thus, asymptomatic cases might not be diagnosed.

Outcomes of *B. procyonis* infection can be severe if diagnosis and treatment are delayed, as was the case for patient B. The patient did not receive a diagnosis for approximately 3 months, resulting in severe neurologic sequelae. A clinician who had encountered a case before treating patient A suspected baylisascariasis in both patients A and B, suggesting that heightened awareness by the health care provider might have contributed to their diagnoses. This clinician’s observation highlights the possibility that similar cases might not be diagnosed without improved awareness among health care providers of the possibility of *B. procyonis* infection.

Epidemiologic investigations suggested the possible exposure of patient A at his residence where a raccoon latrine was present and where he was suspected to have consumed material contaminated with raccoon feces. Additional environmental investigations would be necessary to confirm that the feces at the residence were the source of infection. Identifying a potential source of infection for patient B was not successful; however, his family reported that he regularly placed soil and other objects in his mouth in outdoor areas that might have been frequented by raccoons.

### Implications for Public Health Practice

Given the severity of disease in humans, the high prevalence of *B. procyonis* infection in raccoons, and the proximity of raccoons to humans and pets, *B. procyonis* is a substantial public health concern. To prevent infection, the public should avoid contact with raccoons and their feces, not keep raccoons as pets, ensure that children or persons with developmental disabilities do not place contaminated objects or fingers into their mouths, practice good hand hygiene after outdoor activities, and safely remove raccoon latrines on properties, paying special attention to flat surfaces such as rooftops, decks, tree stumps, or unsealed attics and other areas where raccoons prefer to defecate** (*6*). Property owners should also take measures to prevent raccoon infestations, including eliminating access to sources of food and water; securing trash in tightly closed containers; closing off access to basements, attics, and crawl spaces; and clearing brush and trees away from the property and roof line to discourage raccoons from sleeping or defecating nearby. Sandboxes on properties should also be covered, if possible, when not in use. Dog owners should also prevent their pets from eating raccoon feces and accessing areas with raccoon feces because dogs can also be infected and shed eggs in their feces (*7*). Pets should be treated with a year-round parasite prevention product that contains an intestinal dewormer effective against *B. procyonis* and have fecal examinations for intestinal parasites performed at least annually by a veterinarian, according to existing guidelines.^††^

Improving awareness among the public is critical for reducing the risk for *B. procyonis* infection, especially given the ubiquity of raccoons in urban settings and the challenges associated with raccoon exclusion. For clinicians, education to heighten awareness and increase recognition is needed. Any patients, especially young children or persons with developmental disabilities or pica, who have progressive neurologic deterioration and high peripheral eosinophilia or eosinophilic meningoencephalitis, should be promptly evaluated. A history of exposure to raccoons or their feces is highly suggestive but not necessary. Empiric treatment of baylisascariasis with albendazole should be considered (*6*).

## Footnotes

† 45 C.F.R. part 46, 21 C.F.R. part 56; 42 U.S.C. Sect. 241(d); 5 U.S.C. Sect. 552a; 44 U.S.C. Sect. 3501 et seq.

§ Although dogs can be definitive *B. procyonis* hosts, they shed fewer eggs than do raccoons. The dog was not tested; however, the owners were advised to administer regular deworming treatment.

¶ Raccoon Roundworm (*Baylisascaris* Infection) | LACDPH

**
Preventing Raccoon Roundworm | Raccoon Roundworm (*Baylisascaris* Infection) | CDC

††
*Baylisascaris procyonis* | Guidelines | Companion Animal Parasite Council
